# Supplementary material for: RNA splicing analysis using heterogeneous and large RNA-seq datasets
Source: Nat Commun. 2023 Mar 3;14:1230. doi: 10.1038/s41467-023-36585-y (PMC9984406; doi:10.1038/s41467-023-36585-y)
Supplement: Supplementary file 1 — Supplementary Information [file 41467_2023_36585_MOESM1_ESM.pdf]

Supplementary Information: RNA splicing analysis using heterogeneous and large RNA-seq datasets

Jorge Vaquero-Garcia,[1†] Joseph K. Aicher,[1,4†] San Jewell,[1†] Matthew R. Gazzara,[1†]
Caleb M. Radens,[1] Anupama Jha,[2] Scott S. Norton,[1] Nicholas F. Lahens,[3]
Gregory R. Grant,[1] Yoseph Barash[1,2*]

[1]Department of Genetics, University of Pennsylvania
[2]Department of Computer and Information Science, University of Pennsylvania
[3]Institute for Translational Medicine and Therapeutics, University of Pennsylvania
[4]Division of Human Genetics, Children's Hospital of Philadelphia

[†]Equal contribution as co-first authors.
[*]To whom correspondence should be addressed; e-mail: yosephb@upenn.edu.

Supplementary Methods

Supplementary Note: procedure for bootstrapped readrates from per-position reads

MAJIQ's bootstrapping procedure can be defined as follows. Without loss of generality, consider a single junction. For each RNA-seq read aligned with a split for this junction, we define the read's position relative to the junction (or vice-versa) with a position i and count the number of reads associated with each position, which we call S_i.

These raw readrates include biases that we would like to correct for; in particular, we define an explicit procedure for removing stacks by comparing the number of reads at each position against a Poisson model using the observed readrates at all other positions, which results in a set

of stack-corrected nonzero readrates R_i for $i \in \{1, \ldots, P\}$, where P is the number of nonzero positions after stack removal. These are the observed units for bootstrapping, so to emphasize:

$$
\begin{aligned}
R_i \equiv & \text{\# of RNA-seq reads for } i\text{-th position}, && \text{(observed readrates)} \\
i \in & \{1, \ldots, P\}. && \text{(nonzero positions after stack removal)}
\end{aligned}
$$

Other methods typically sum directly over positions R_i (really S_i since they generally also ignore read stacks) to produce a total junction readrate for use in quantification:

$$
R \equiv \sum_{i=1}^{P} R_i. \qquad \text{(observed total junction readrate)}
$$

Since we are unsatisfied with uncertainty/variance accounted for by directly using R, we generate samples from a bootstrap distribution over the P nonzero positions.

If we make the assumption that we are given the number of nonzero positions P and that the underlying readrate for each of these positions is independent and identically distributed with finite mean $\mathbb{E}[R_i] = \mu$ and variance $\mathbb{V}[R_i] = \sigma^2$, we can derive the mean and variance of our observed total readrate:

$$
\begin{aligned}
\mathbb{E}[R] =& \mathbb{E}\left[\sum_{i=1}^{P} R_i\right] \\
=& \sum_{i=1}^{P} \mathbb{E}[R_i] \\
=& \mu P, && \text{(observed total readrate mean)} \\
\mathbb{V}[R] =& P\sigma^2. && \text{(observed total readrate variance)}
\end{aligned}
$$

If we were able to take two samples for the observed total readrate (i.e. R and R'), their difference has mean 0 and variance $2P\sigma^2$.

We define our bootstrapping procedure over observed nonzero reads $R_1, \ldots, R_P$ to generate bootstrapped total reads $\hat{R}, \hat{R}', \ldots$ such that the variance of the difference between bootstrap samples would be equivalent to that of the difference between two samples from the true distribution (i.e. $2P\sigma^2$). In order to do this, we take $P - 1$ samples from $\{R_1, \ldots, R_P\}$ with replacement and scale their sum by $P/(P - 1)$.

It is straightforward to see that the bootstrapped total readrate has the same mean as the observed total readrate. In order to prove that the variance of the difference between two sample matches, we note that the covariance $\mathrm{Cov}\left(R_{Z_k}, R_{Z_{k'}}\right)$ between any two draws from the observed per-position readrates with $Z_i \sim \mathrm{Uniform}(P)$ is:

$$\begin{aligned}\mathrm{Cov}\left(R_{Z_k}, R_{Z_{k'}}\right) =& \mathbb{E}\left[\left(R_{Z_k} - \mu\right)\left(R_{Z_{k'}} - \mu\right)\right] \\ =& \mathbb{E}\left[R_{Z_k} R_{Z_{k'}}\right] - \mu^2.\end{aligned}$$

. We note that $\mathbb{E}\left[R_i R_j\right] = \sigma^2 \delta_{ij} + \mu^2$ (where δ_{ij} is the Kroencker delta). When $k = k'$, it follows that $\mathbb{E}\left[R_{Z_k} R_{Z_{k'}}\right] = \sigma^2 + \mu^2$. Otherwise, the law of total expectation gives:

$$\begin{aligned}\mathbb{E}\left[R_{Z_k} R_{Z_{k'}}\right] =& \mathbb{E}\left[\mathbb{E}\left[R_{Z_k} R_{Z_{k'}} \middle| Z_k, Z_{k'}\right]\right] && \text{(given } k \neq k'\text{)} \\ =& \frac{1}{P^2} \sum_{i=1}^{P} \sum_{j=1}^{P} \sigma^2 \delta_{ij} + \mu^2 \\ =& \mu^2 + \frac{1}{P}\sigma^2.\end{aligned}$$

Combining the two cases, we have:

$$\begin{aligned}\mathbb{E}\left[R_{Z_k} R_{Z_{k'}}\right] =& \delta_{kk'}\left(\mu^2 + \sigma^2\right) + \left(1 - \delta_{kk'}\right)\left(\mu^2 + \frac{1}{P}\sigma^2\right) \\ =& \mu^2 + \frac{1}{P}\sigma^2 + \delta_{kk'}\frac{P-1}{P}\sigma^2. && \text{(second moment sampled readrate)}\end{aligned}$$

Therefore,

$$\mathrm{Cov}\left(R_{Z_k}, R_{Z_{k'}}\right) = \frac{1}{P}\sigma^2 + \delta_{kk'}\frac{P-1}{P}\sigma^2. \qquad \text{(covariance sampled readrate)}$$

Thus, the variance of the bootstrapped total readrate is

$$\begin{aligned}
\mathbb{V}\left[\hat{R}\right] =& \frac{P^2}{(P-1)^2}\mathbb{V}\left[\sum_{k=1}^{P-1} R_{Z_k}\right] \\
=& \frac{P^2}{(P-1)^2}\sum_{k=1}^{P-1}\sum_{k'=1}^{P-1} \operatorname{Cov}\left(R_{Z_k}, R_{Z_{k'}}\right) \\
=& \frac{P^2}{(P-1)^2}\sum_{k=1}^{P-1}\sum_{k'=1}^{P-1} \frac{1}{P}\sigma^2 + \delta_{kk'}\frac{P-1}{P}\sigma^2 \\
=& 2P\sigma^2. && \text{(true bootstrap readrate variance)}
\end{aligned}$$

But we want the variance of the difference between two samples from the bootstrap procedure. So, we calculate the covariance between two distinct samples:

$$\begin{aligned}
\operatorname{Cov}\left(\hat{R}, \hat{R}'\right) =& \frac{P^2}{(P-1)^2}\sum_{k=1}^{P-1}\sum_{k'=1}^{P-1} \frac{1}{P}\sigma^2 \\
=& P\sigma^2. && \text{(covariance between samples of } \hat{\mathcal{P}}\text{)}
\end{aligned}$$

Therefore, we find that:

$$\begin{aligned}
\mathbb{V}\left[\hat{R} - \hat{R}'\right] =& 2\mathbb{V}\left[\hat{R}\right] - 2\operatorname{Cov}\left(\hat{R}, \hat{R}'\right) \\
=& 4P\sigma^2 - 2P\sigma^2 \\
=& 2P\sigma^2. && \text{(bootstrap total readrate variance as difference)}
\end{aligned}$$

In practice, the observed nonzero positions can lead to a bootstrap distribution with variance less than its mean (underdispersed). We generally expect readrates to follow a Poisson or negative binomial (overdispersed) distribution, so in these cases, we fall back to parametric bootstrapping with a Poisson distribution with mean μP. Otherwise, we use the nonparametric bootstrap sampling procedure as described above.

Supplementary Figures

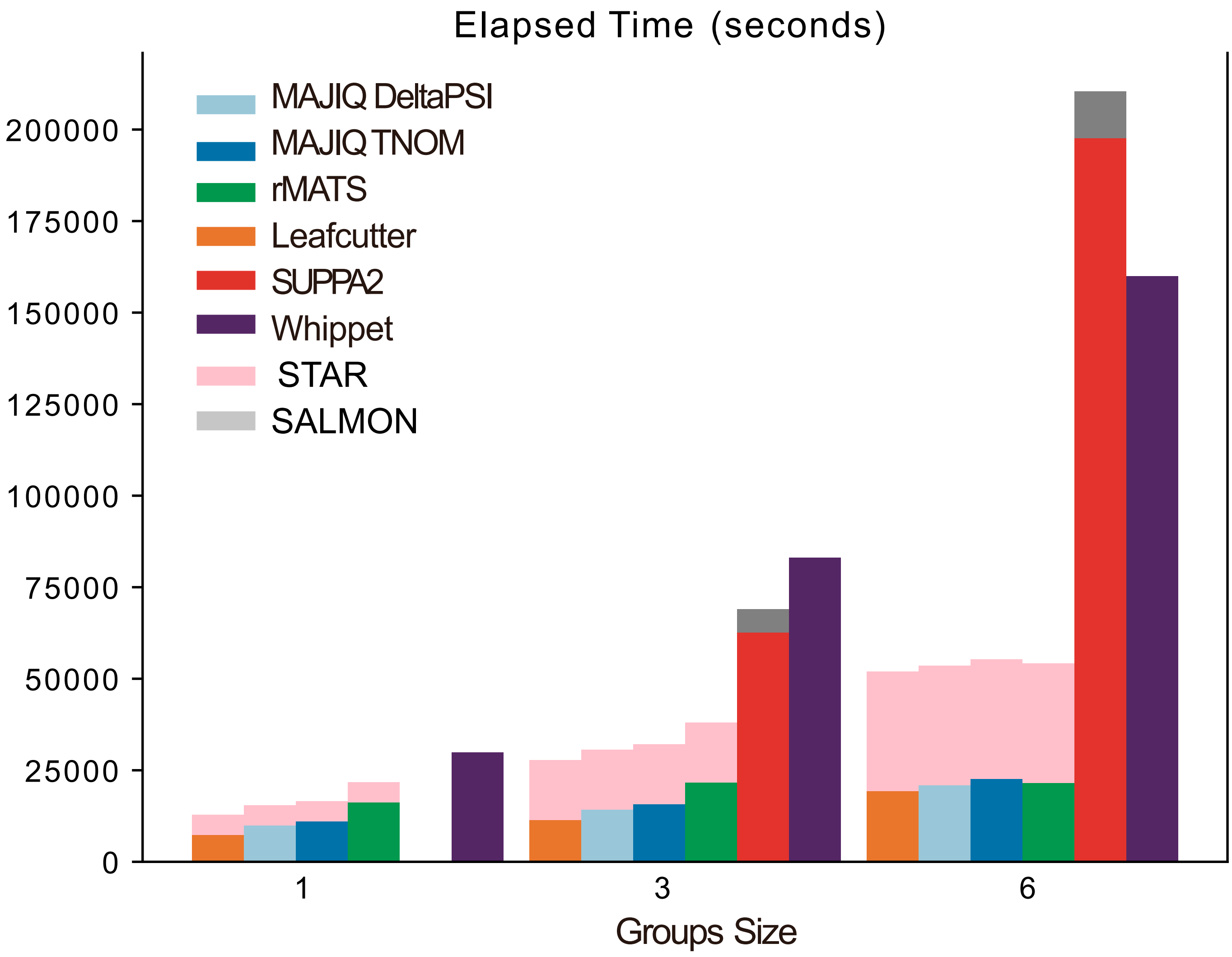

Supplementary Fig. 1: Run time analysis without parallelization script Analysis of running time on a 16 core desktop machine, same as in Fig. 2a, but using all software 'as is' without adding user parallelization scripts for Whippet and SUPPA2.

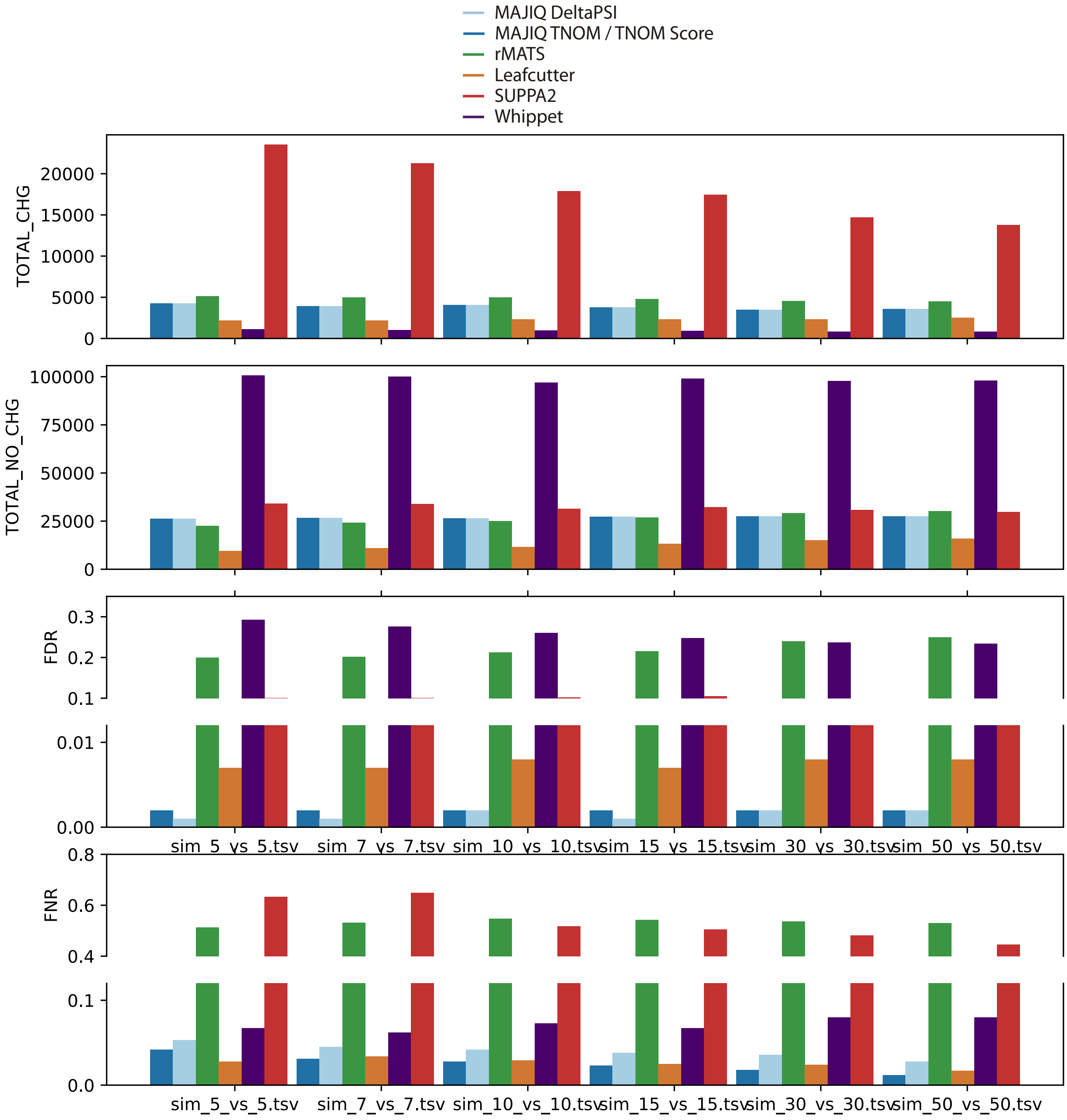

Supplementary Fig. 2: Performance evaluation using simulated data at event level. This figure is equivalent to Fig. 2b in the main text but displays the results when using each method's unique event definition rather than aggregated at the gene level. For methods that quantify local

AS events such as rMATS and MAJIQ, the number of changing events is approximately double that of changing genes (2,337 vs 4,267 for MAJIQ HET), while for LeafCutter, which uses a coarser definition for events based on intron clusters, the number of changing events and genes is similar (1,739 vs 2,169).

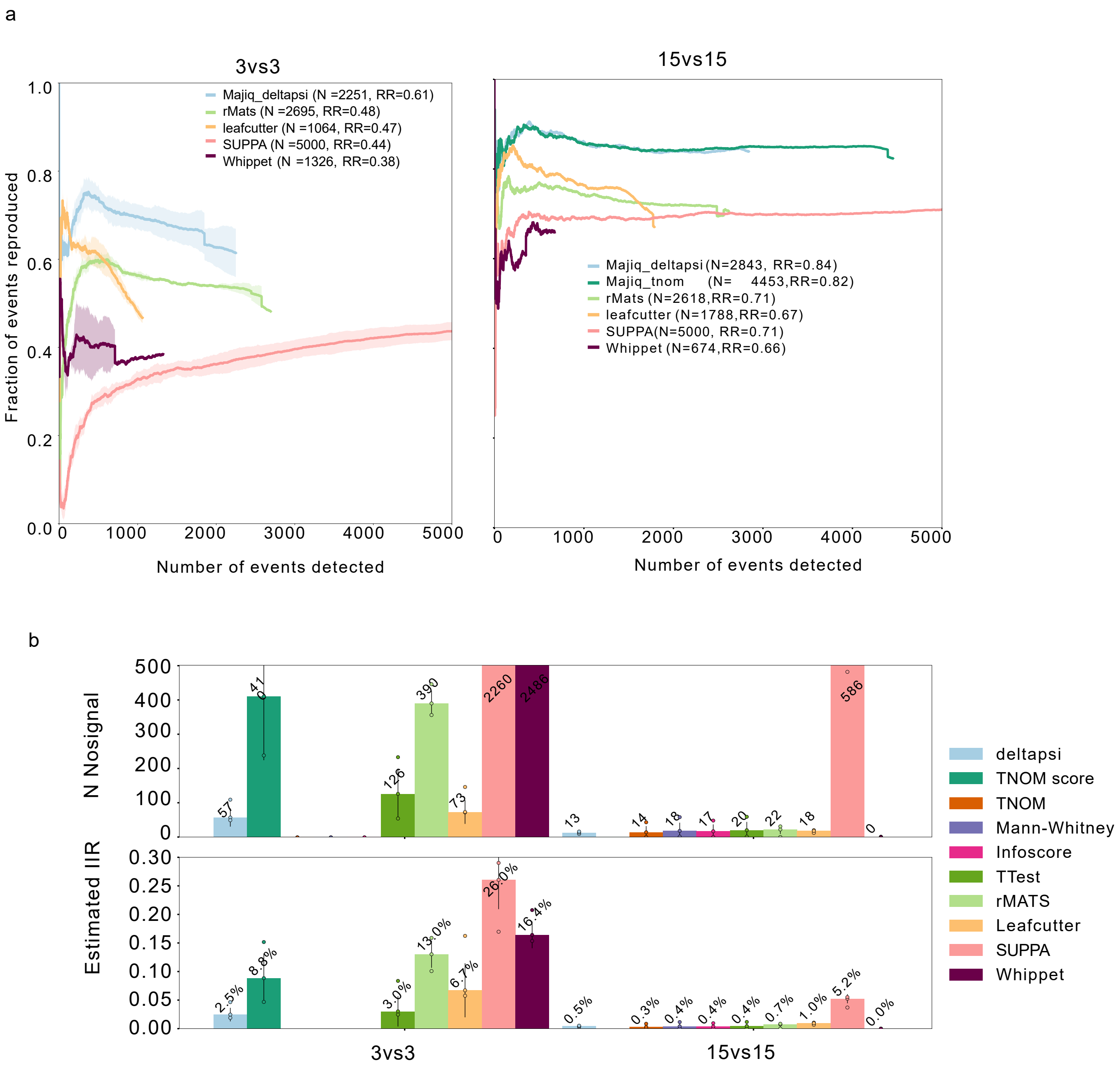

Supplementary Fig. 3: Robustness of RR and IIR results across multiple sample sets. This figure is equivalent to Fig. 2d,e in the main text but displays the results of RR (a, cerebellum vs. muscle, top) and IIR (b, cerebellum vs. cerebellum, bottom) when using three different randomly selected subsets of samples for groups of size 3 (left) and 15 (right). Solid lines in (a) and bar heights in (b) represent the mean of each group and shaded region in (a) and error bars in (b) represent plus or minus one standard deviation.

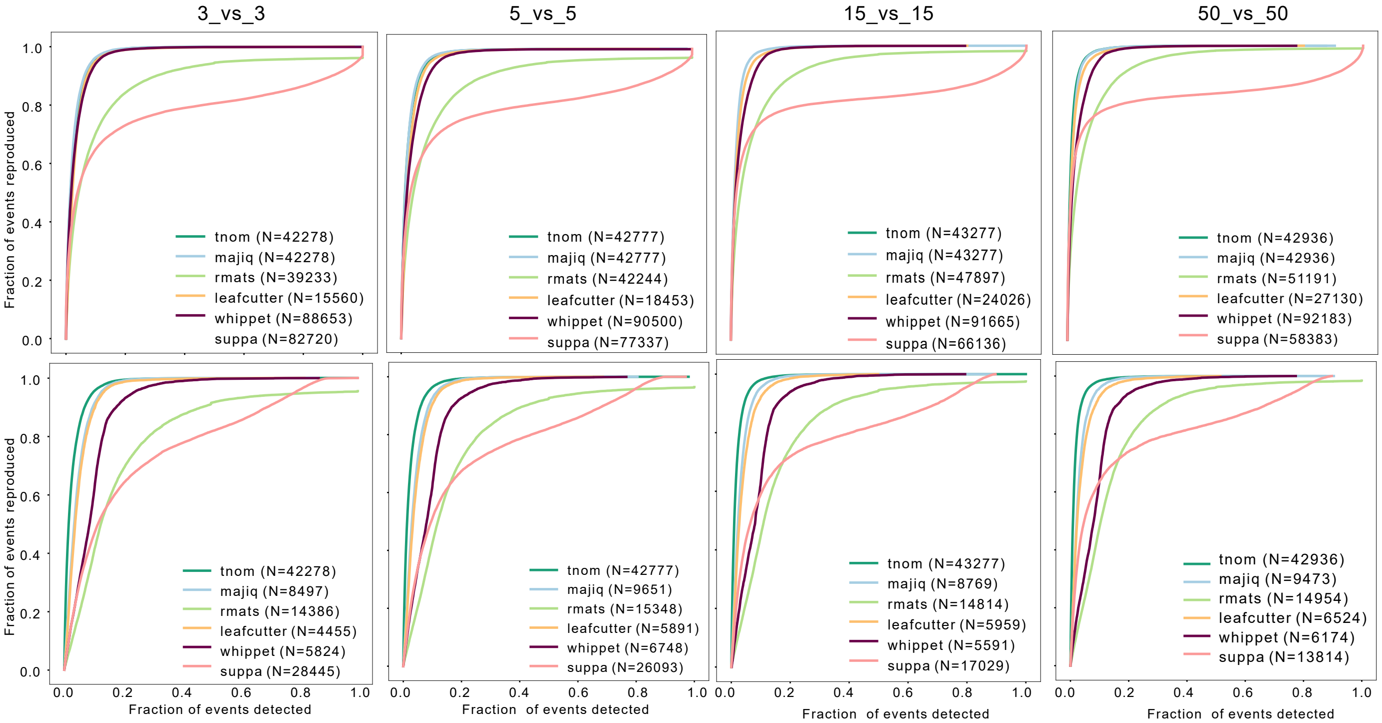

Supplementary Fig. 4: dPSI accuracy. Here we assess each method's dPSI accuracy irrespective of the statistical test. Each event reported as exhibiting a change of dPSI $> 10\%$ (top) or dPSI $> 20\%$ (bottom) is compared against the synthetic data ground truth (same GTEX based synthetic data as in Fig2b) and the CDF over absolute deviation between computed and true dPSI is plotted. Numbers in the legend represent the total number of events reported by each method. Columns represent the size of the groups compared (3, 5, 15, 50).

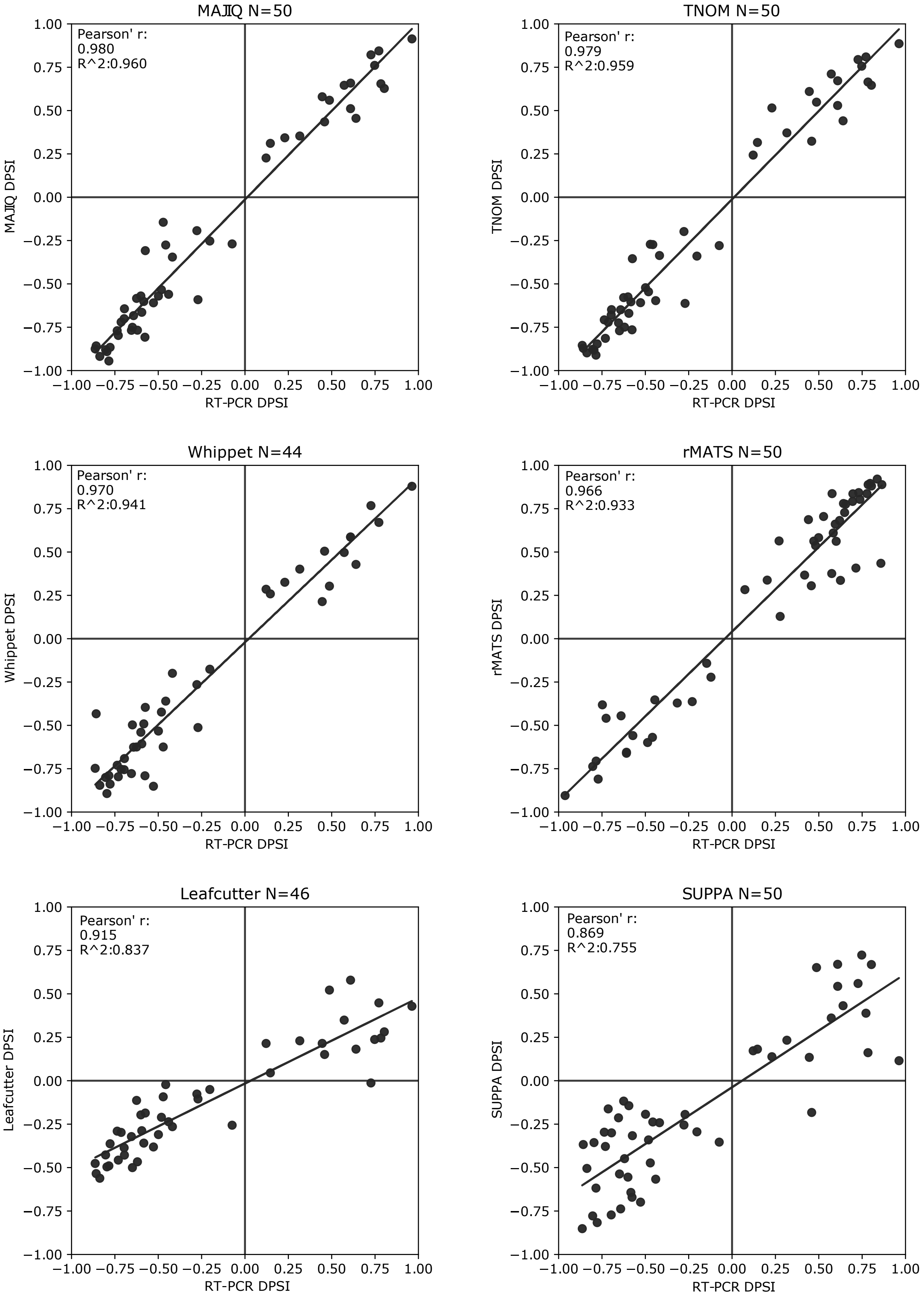
MAJIQ N=50
Pearson' r: 0.980 R^2:0.960
MAJIQ DPSI
RT-PCR DPSI
TNOM N=50
Pearson' r: 0.979 R^2:0.959
TNOM DPSI
RT-PCR DPSI
Whippet N=44
Pearson' r: 0.970 R^2:0.941
Whippet DPSI
RT-PCR DPSI
rMATS N=50
Pearson' r: 0.966 R^2:0.933
rMATS DPSI
RT-PCR DPSI
Leafcutter N=46
Pearson' r: 0.915 R^2:0.837
Leafcutter DPSI
RT-PCR DPSI
SUPPA N=50
Pearson' r: 0.869 R^2:0.755
SUPPA DPSI
RT-PCR DPSI

Supplementary Fig. 5: Correlation between splicing quantification algorithms and RT-PCR. Correlation between RNA-seq based dPSI calculated by various differential splicing quantification algorithms (y-axis) and RT-PC based dPSI comparing mouse liver with mouse cerebellum (x-axis) in triplicate. RT-PCR quantifications are from (*1*) using RNA extracted by (*2*) to produce the matching RNA-seq samples. Note that all splicing events shown here were selected by (*1*) to be binary, annotated, and changing between the two tissues to allow direct comparison to rMATS. The usage of simple binary events allowed us to calibrate LeafCutter's intron cluster quantifications to PSI (range is $|0 - 0.5|$ as the two inclusion junctions are being considered separately by LeafCutter), which is not possible in the general case.

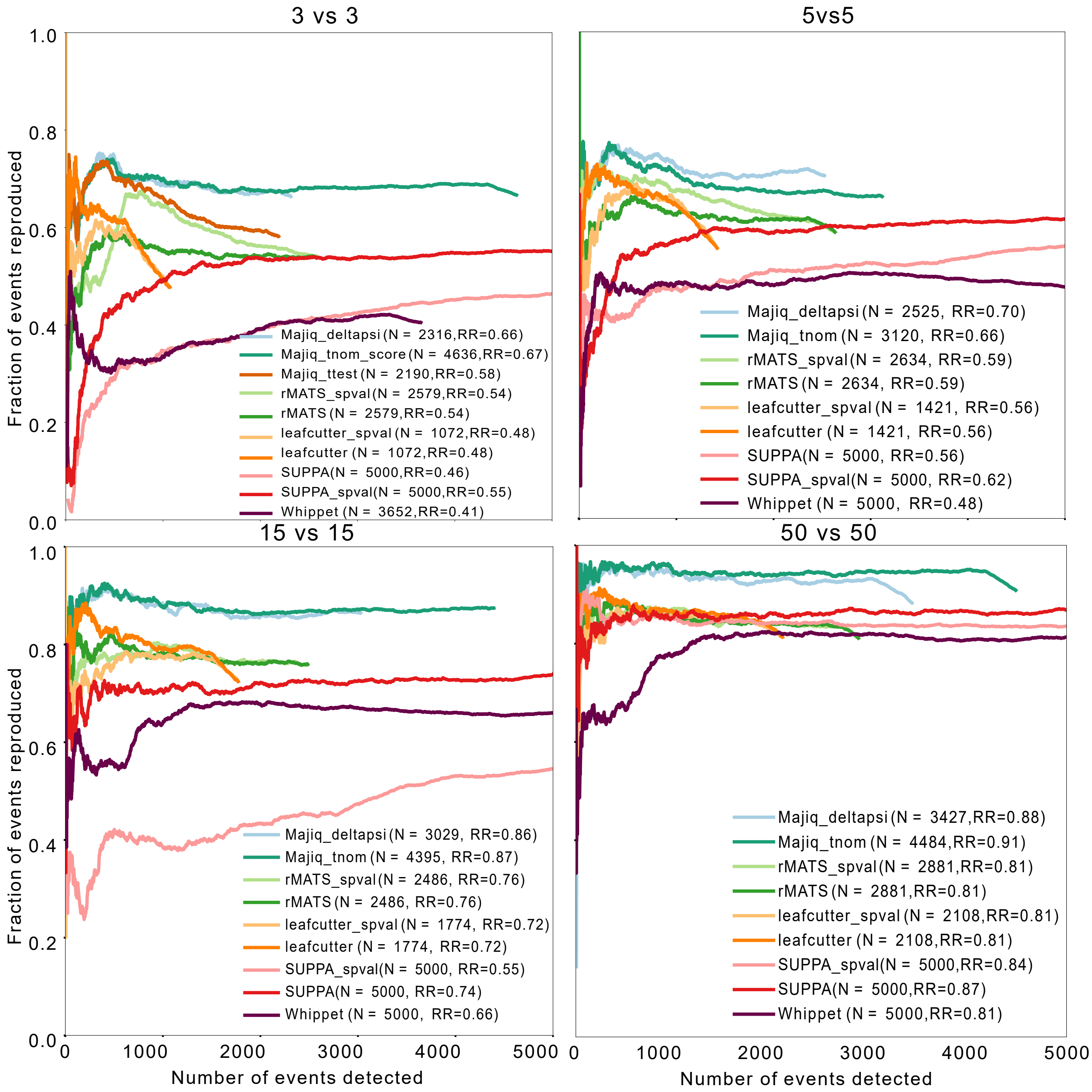

Supplementary Fig. 6: Reproducibility ratio plots ranking by p-value or dPSI. Most methods provides both a p-value and dPSI, and they are used to filter the events, but how to rank the resulting set of events is not defined by the authors. We assessed the effect of ranking first by each tool's p-value or first by dPSI on the resulting RR plots for different group sizes (3, 5, 15, 50). In all cases we find ranking by dPSI, as used in main Fig 2d, to gives better results.

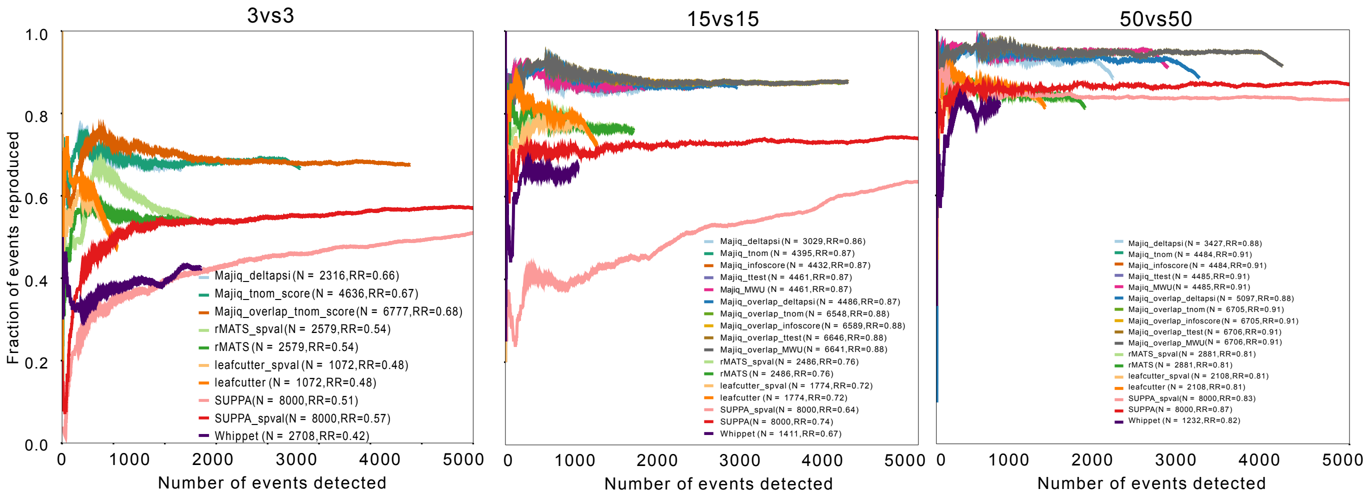

Supplementary Fig. 7: Reproducibility ratio plots without filtering MAJIQ overlapping LSV. This figure is equivalent to Fig. 2d in the main text but displays the RR results when no filtering of overlapping LSVs is performed in MAJIQ. Note the RR curves remain stable but the total number of events reported is inflated by approximately 50%. Similar to Supplementary Fig. 6 pval indicates when each tool pval is used as ranking method (See methods). MAJIQ HET test abbreviations are: tnom, Total Number of Misclassifications; MWU, Mann-Whitney U test.

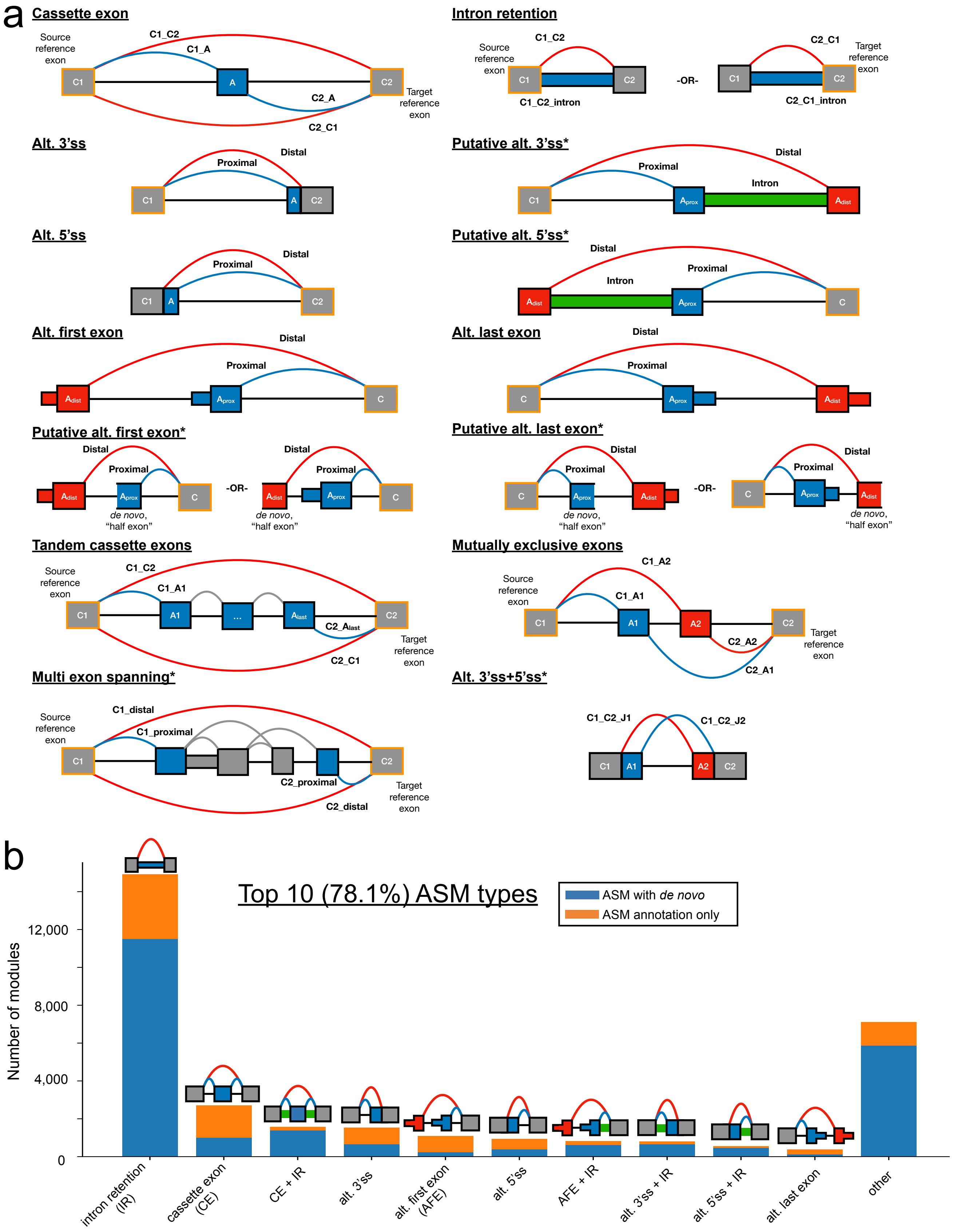
a
Cassette exon
Source reference exon
C1_C2
C1_A
C1
A
C2
C2_A
Target reference exon
C2_C1
Intron retention
C1_C2
Source reference exon
C1
C2
C1_C2_intron
C2_C1
Target reference exon
C2_C1_intron
Alt. 3'ss
Distal
Proximal
Putative alt. 3'ss*
Intron
Aprox
Adist
Alt. 5'ss
Putative alt. 5'ss*
Alt. first exon
Alt. last exon
Putative alt. first exon*
de novo, "half exon"
Putative alt. last exon*
Tandem cassette exons
C1_A1
A1
Alast
C2_Alast
Mutually exclusive exons
C1_A2
A2
C2_A2
C2_A1
Multi exon spanning*
C1_distal
C1_proximal
C2_proximal
C2_distal
Alt. 3'ss+5'ss*
C1_C2_J1
C1_C2_J2
b
Top 10 (78.1%) ASM types
ASM with de novo
ASM annotation only
Number of modules
0
4,000
8,000
12,000
intron retention (IR)
cassette exon (CE)
CE + IR
alt. 3'ss
alt. first exon (AFE)
alt. 5'ss
AFE + IR
alt. 3'ss + IR
alt. 5'ss + IR
alt. last exon
other

Supplementary Fig. 8: VOILA Modulizer AS event types. **(a)** Diagrams outlining the structure of alternative splicing event (AS event) types used in the VOILA Modulizer. Exons and junctions are labeled in a way consistent with the tab separated value text file outputs of the Modulizer. Gray exons outlined in orange indicate the reference exon(s) from local splicing variations (LSVs, source and/or target) used to create the splicing events. Blue junctions, introns, exons, and exonic regions correspond to inclusion products while red corresponds to exclusion products. Gray junctions in tandem cassette exons and multi-exon skipping correspond to other junctions present in the splicegraph after simplification, but are not directly considered or output by the Moduilizer in terms of quantifications. Green introns in putative $5'$ and $3'$ss events indicate a retained intron that was quantified to high inclusion, but had the corresponding splice junction removed during simplification due to low PSI. This suggests $\mathrm{A_{prox}}$, the intron, and $\mathrm{A_{dist}}$ behave as a single exon unit with the red (intron distal) and blue (intron proximal) splice junctions acting as alternative splice sites. Asterisks indicate non-classical AS event types. **(b)** Stacked barchart showing the AS event makeup of the top 10 alternative splicing modules (ASMs) across the 13 GTEx brain tissue groups from the VOILA Modulizer after applying a 5% PSI simplification threshold (e.g. junctions with a group median of less than 5% in all groups are removed). Modules named with a plus sign (e.g. CE + IR) correspond to AS modules made up of more than one AS event type (e.g. CE + IR modules were made up of both cassette exon and intron retention events). Blue bar regions indicate AS modules that had one or more de novo or unannotated junctions, after simplification, while orange regions indicate AS modules consisting of solely annotated junctions and/or retained introns.

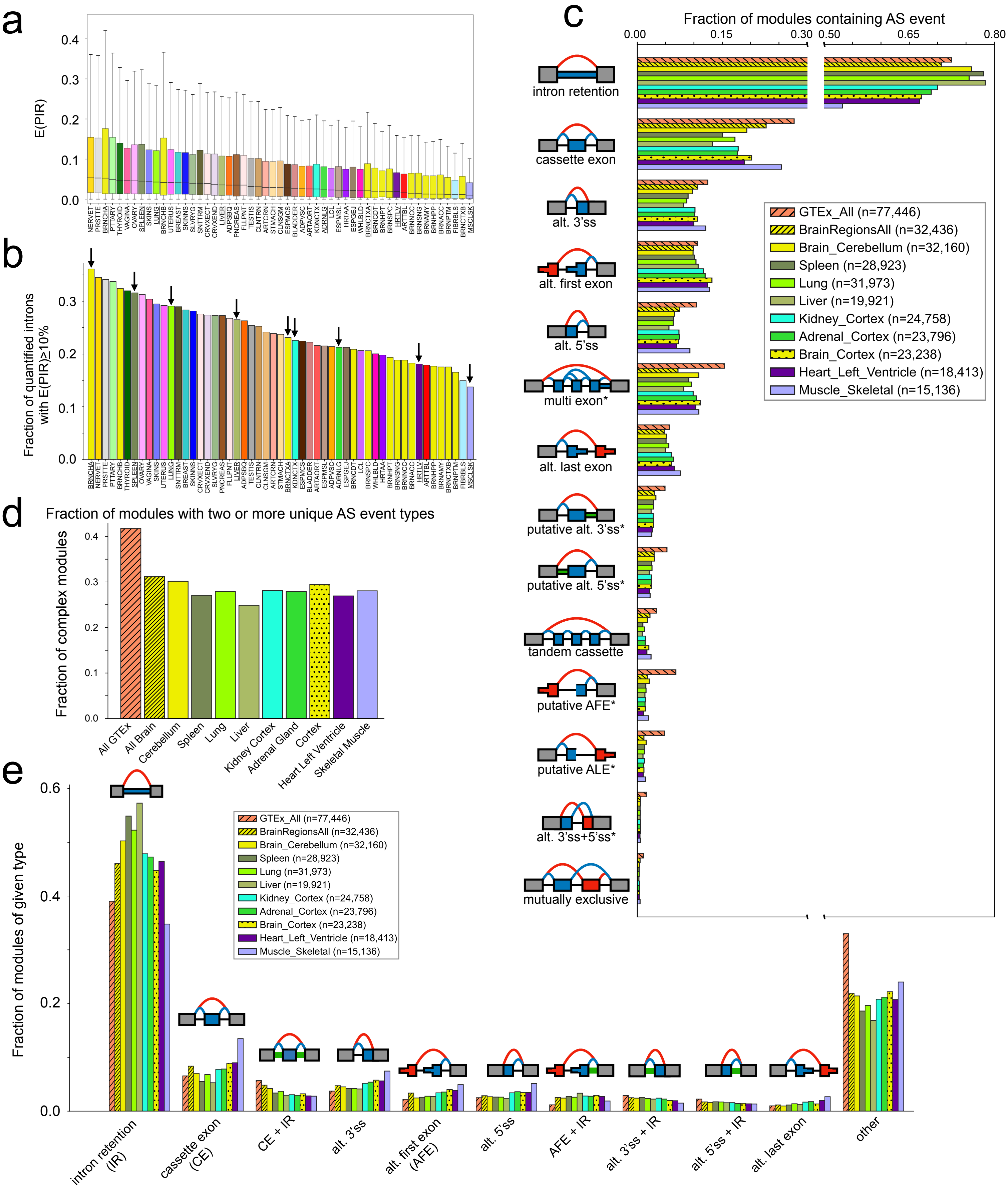
a
E(PIR)
b
Fraction of quantified introns with E(PIR)≥10%
c
Fraction of modules containing AS event
intron retention
cassette exon
alt. 3'ss
alt. first exon
alt. 5'ss
multi exon*
alt. last exon
putative alt. 3'ss*
putative alt. 5'ss*
tandem cassette
putative AFE*
putative ALE*
alt. 3'ss+5'ss*
mutually exclusive
GTEx_All (n=77,446)
BrainRegionsAll (n=32,436)
Brain_Cerebellum (n=32,160)
Spleen (n=28,923)
Lung (n=31,973)
Liver (n=19,921)
Kidney_Cortex (n=24,758)
Adrenal_Cortex (n=23,796)
Brain_Cortex (n=23,238)
Heart_Left_Ventricle (n=18,413)
Muscle_Skeletal (n=15,136)
d
Fraction of modules with two or more unique AS event types
Fraction of complex modules
All GTEx
All Brain
Cerebellum
Spleen
Lung
Liver
Kidney Cortex
Adrenal Gland
Cortex
Heart Left Ventricle
Skeletal Muscle
e
Fraction of modules of given type
intron retention (IR)
cassette exon (CE)
CE + IR
alt. 3'ss
alt. first exon (AFE)
alt. 5'ss
AFE + IR
alt. 3'ss + IR
alt. 5'ss + IR
alt. last exon
other

Supplementary Fig. 9: Intron retention, AS events, and AS module types across GTEx tissues. **(a)** Boxplots of distribution of Percent Intron Retention (E(PIR)) for unique, quantified introns across all GTEx tissues. Quantifications are based on a subset of 15 samples for each tissue using MAJIQ PSI quantification (see Methods). In cases where the same intron or intronic fragment was quantified multiple times (i.e. source and target LSV), the maximum value was retained. Colors and abbreviations are consistent with those used in the GTEx publication (*3*) with yellow indicating brain tissues. For each tissue, boxes represent the 25th and 75th percentiles, black lines represent the median, and whiskers represent 1.5 times the interquartile range for E(PIR) values. **(b)** Barplot displaying the fraction of quantified introns in each tissue with E(PIR)$> 10\%$ with labels as described above. Arrowheads indicate tissues that were analyzed with the Modulizer to define AS events and AS modules. **(c)** Clustered bar chart showing the fraction of AS modules that contain each of the binary AS event types defined by running the Modulizer on all 53 GTEx tissue groups (orange hatched), all 13 brain tissues (yellow hatched), or on specific tissues (plain colors) after applying a 5% E(PSI) simplification threshold. Colors and number of AS modules in each tissue are defined in the inset. **(d)** Bar chart showing the fraction of complex AS modules (those made up of two or more distinct AS event types) from GTEx tissue groups (line-hatched bars) or individual tissues listed. Number of AS modules and colors are defined by the inset in panel c. **(e)** Clustered bar chart showing the fraction of total AS modules belonging to each of the top 10 most common AS module types defined by the brain subregion analysis in Supplementary Fig. 8b. AS modules were defined at a tissue group level (line-hatched bars) or within individual tissues after applying the 5% E(PSI) simplification. Modules named with a plus sign (e.g. CE + IR) correspond to AS modules made up of more than one AS event type (e.g. CE + IR modules were made up of both cassette exon and intron retention events). Colors and number of AS modules in each tissue are defined in the inset.

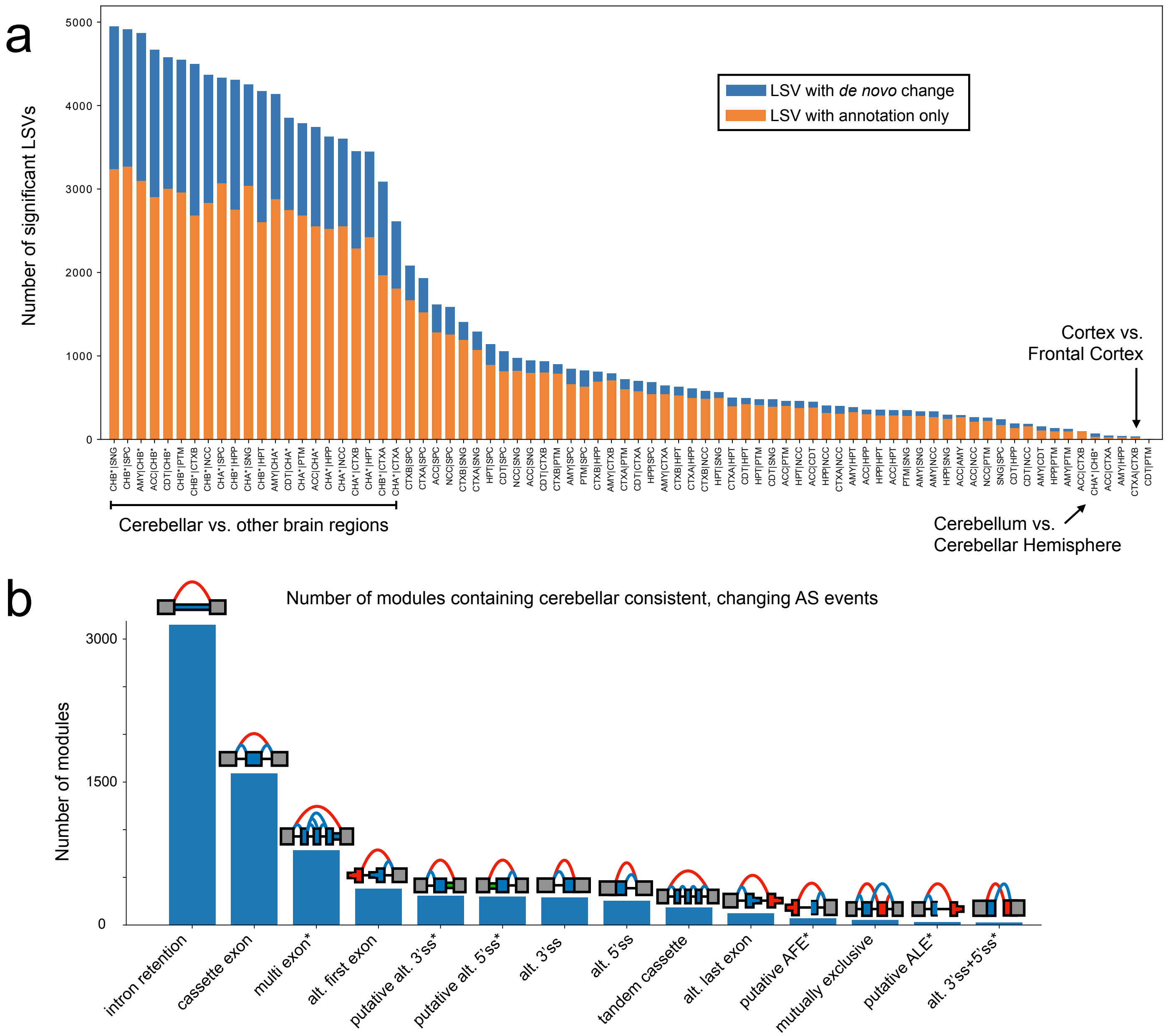
a
Number of significant LSVs
5000
4000
3000
2000
1000
0
LSV with *de novo* change
LSV with annotation only
Cortex vs.
Frontal Cortex
CHB*|SNG
CHB*|SPC
AMY|CHB*
ACC|CHB*
CDT|CHB*
CHB*|PTM
CHB*|CTXB
CHB*|NCC
CHA*|SPC
CHB*|HPP
CHA*|SNG
CHB*|HPT
AMY|CHA*
CDT|CHA*
CHA*|PTM
ACC|CHA*
CHA*|HPP
CHA*|NCC
CHA*|CTXB
CHA*|HPT
CHB*|CTXA
CHA*|CTXA
CTXB|SPC
CTXA|SPC
ACC|SPC
NCC|SPC
CTXB|SNG
CTXA|SNG
HPT|SPC
CDT|SPC
NCC|SNG
ACC|SNG
CDT|CTXB
CTXB|PTM
AMY|SPC
PTM|SPC
CTXB|HPP
AMY|CTXB
CTXA|PTM
CDT|CTXA
HPP|SPC
AMY|CTXA
CTXB|HPT
CTXA|HPP
CTXB|NCC
HPT|SNG
CTXA|HPT
CDT|HPT
HPT|PTM
CDT|SNG
ACC|PTM
HPT|NCC
ACC|CDT
HPP|NCC
CTXA|NCC
AMY|HPT
ACC|HPP
HPP|HPT
ACC|HPT
PTM|SNG
AMY|SNG
AMY|NCC
HPP|SNG
ACC|AMY
ACC|NCC
NCC|PTM
SNG|SPC
CDT|HPP
CDT|NCC
AMY|CDT
HPP|PTM
AMY|PTM
ACC|CTXB
CHA*|CHB*
ACC|CTXA
AMY|HPP
CTXA|CTXB
CDT|PTM
Cerebellar vs. other brain regions
Cerebellum vs.
Cerebellar Hemisphere
b
Number of modules containing cerebellar consistent, changing AS events
Number of modules
3000
1500
0
intron retention
cassette exon
multi exon*
alt. first exon
putative alt. 3'ss*
putative alt. 5'ss*
alt. 3'ss
alt. 5'ss
tandem cassette
alt. last exon
putative AFE*
mutually exclusive
putative ALE*
alt. 3'ss+5'ss*

Supplementary Fig. 10: Cerebellar vs other brain tissues with LSVs and splicing modules. **(a)** Barchart showing the number of significant LSVs from 78 pairwise MAJIQ HET comparisons between the 13 GTEx brain tissue groups. Significant LSVs were those containing at least one junction or intron with an absolute difference in group median expected PSI values of 20% between two tissue groups and all four HET statistics (Mann-Whitney, InfoScore, TNOM, and t-test) with two-sided $p < 0.05$. Comparisons that include a cerebellar tissue (Cerebellum, CHA; or Cerebellar Hemisphere, CHB) are highlighted. Blue indicates LSVs containing an unannotated, de novo junction/intron that was changing and orange indicates LSVs with only annotated junctions/introns that changed. **(b)** Barchart showing the number of modules containing at least one of the 14 alternative splicing event types found to be significantly changing in both cerebellar tissues versus one or more other brain subregion tissues in a consistent way (see Methods). Event types are outlined in Supplementary Fig. 8a. Non-classical event types are marked with an asterisk.

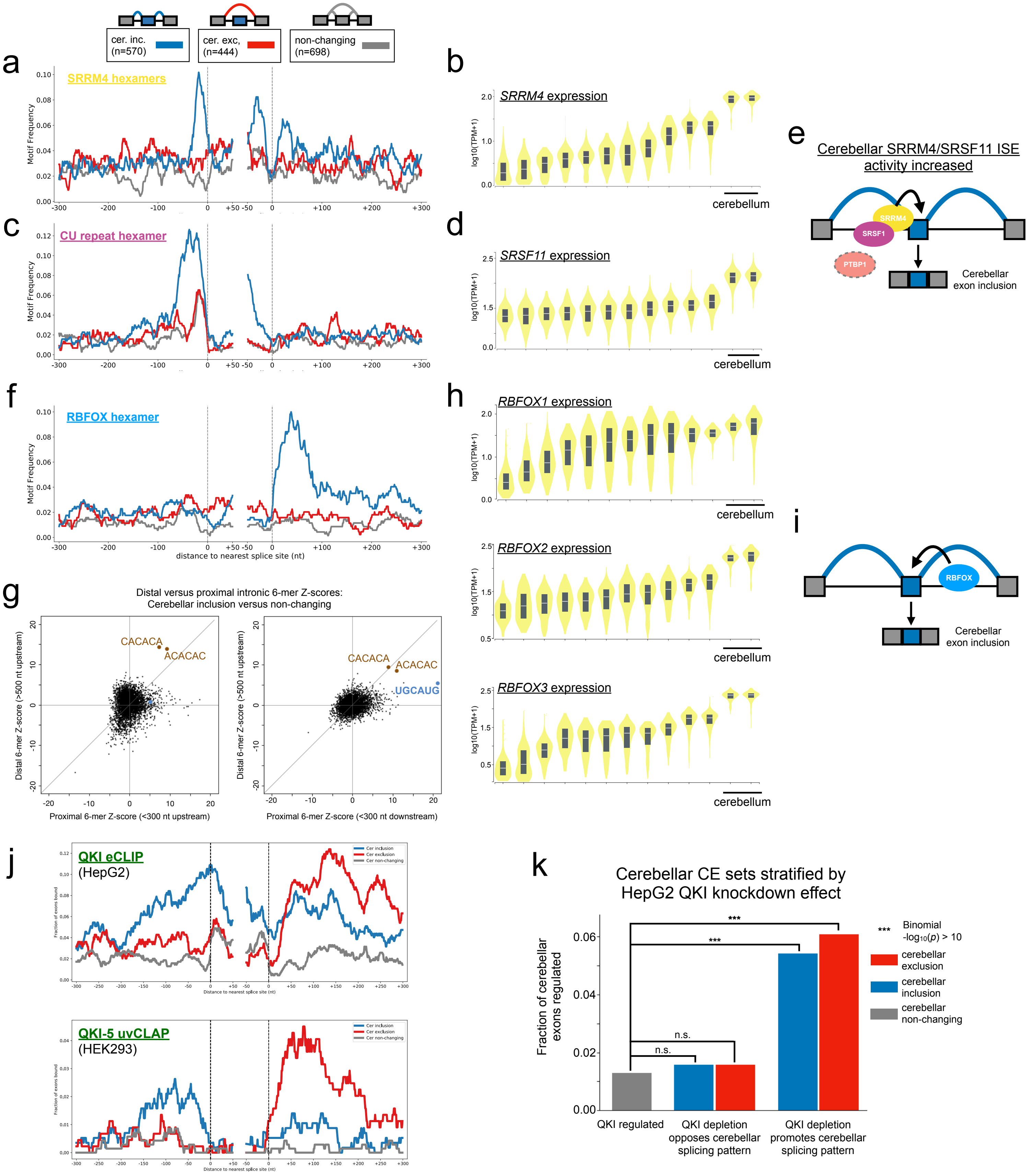

cer. inc. (n=570)
cer. exc, (n=444)
non-changing (n=698)
a
SRRM4 hexamers
Motif Frequency
b
SRRM4 expression
log10(TPM+1)
cerebellum
c
CU repeat hexamer
d
SRSF11 expression
e
Cerebellar SRRM4/SRSF11 ISE activity increased
SRRM4
SRSF1
PTBP1
Cerebellar exon inclusion
f
RBFOX hexamer
distance to nearest splice site (nt)
h
RBFOX1 expression
RBFOX2 expression
RBFOX3 expression
i
RBFOX
Cerebellar exon inclusion
g
Distal versus proximal intronic 6-mer Z-scores: Cerebellar inclusion versus non-changing
CACACA
ACACAC
UGCAUG
Distal 6-mer Z-score (>500 nt upstream)
Proximal 6-mer Z-score (<300 nt upstream)
Proximal 6-mer Z-score (<300 nt downstream)
j
QKI eCLIP
(HepG2)
QKI-5 uvCLAP
(HEK293)
Cer inclusion
Cer exclusion
Cer non-changing
Fraction of exons bound
Distance to nearest splice site (nt)
k
Cerebellar CE sets stratified by HepG2 QKI knockdown effect
Fraction of cerebellar exons regulated
*** Binomial -log10(p) > 10
n.s.
cerebellar exclusion
cerebellar inclusion
cerebellar non-changing
QKI regulated
QKI depletion opposes cerebellar splicing pattern
QKI depletion promotes cerebellar splicing pattern

Supplementary Fig. 11: motif enrichment and RBP expression for changing and non-changing cassette exons. **(a)** RNAmaps showing the frequency of the top UGC containing SRRM4/nSR100 hexamer motifs, as determined by iCLIP (UGCUGC, CUGCUG, GCUGCC, GCUGCU (*4*)), around cerebellar inclusion (blue), exclusion (red), or non-changing (gray) CEs. Frequency was determined by searching for motif occurrence over sliding windows of 20 nucleotides with smoothing using a running mean of 5 nucleotides. **(b)** GTEx *SRRM4* bulk brain tissue gene expression ($\log_{10}(1 + \mathrm{TPM})$) sorted by median. Chart generated using `gtexportal.org` where violins represent the distribution of values, boxes represent the 25th and 75th percentiles, white line represents median, and outlier points outside 1.5 times the interquartile range are shown. Each tissue is represented by no fewer than 109 RNA-seq samples. **(c)** RNAmaps showing the frequency of the top CU-repeat hexamers that bind SRSF11, as determined by iCLIP (UCUCUC and CUCUCU (*5*)), around cerebellar inclusion (blue), exclusion (red), or non-changing (gray) CEs. Frequency was determined by searching for motif occurrence over sliding windows of 20 nucleotides with smoothing using a running mean of 5 nucleotides. **(d)** GTEx *SRSF11* bulk brain tissue gene expression as defined in (b). **(e)** Model for SRRM4/SRSF11 promotion of exon inclusion in cerebellar tissues. Increased expression of SRRM4 and SRSF11 increases intronic splicing enhancer (ISE) activity by increased binding to CU- and UGC- rich regions just upstream of cerebellar included exons. Decreased PTB expression, which also binds CU repeat elements (*6*), may also contribute to increased SRSF11 activity. Model is based on previous work showing cooperative binding and splicing enhancement of neuronal microexons by SRSF11 and SRRM4 (*5*). **(f)** RNAmap showing the frequency of the RBFOX hexamer, UGCAUG, around cerebellar inclusion (blue), exclusion (red), or non-changing (gray) CEs. Frequency was determined by searching for motif occurrence over sliding windows of 20 nucleotides with smoothing using a running mean of 5 nucleotides. **(g)** Scatter plots showing hexamer Z-score correspondence between intronic regions proximal

(<300 nucleotides) or distal (>500 nucleotides) to cerebellar inclusion cassette exons either upstream (left) or downstream (right). CA repeat motifs and the RBFOX hexamer are highlighted. **(h)** GTEx RBFOX family bulk brain tissue gene expression ($\log_{10}(1 + \mathrm{TPM})$) for *RBFOX1*, *RBFOX2*, and *RBFOX3* as defined in (b). **(i)** Model for position dependent RBFOX regulation in GTEx brain tissues. Increased expression of RBFOX family members in cerebellar tissues leads to increased intronic splicing enhancer activity (ISE) through increased RBFOX binding downstream of exons, resulting in cerebellar exon inclusion (blue), when compared to other brain tissue groups. **(j)** RNAmaps showing the frequency of QKI CLIP peak occurrence, indicating *in vivo* binding of QKI around cerebellar inclusion (blue), exclusion (red), or non-changing (gray) CEs. Top plot shows the frequency of QKI eCLIP peaks in HepG2 cells (*7*) while bottom shows uvCLAP peak frequencies for the predominantly nuclear isoform of QKI (QK-5) in HEK293 cells that is thought to regulate splicing (*8*). **(k)** Fraction of given cerebellar cassette exon sets that showed QKI regulation (determined by shRNA knockdown of QKI in HepG2 cells, see Methods) where the effect of QKI depletion either promoted cerebellar splicing patterns (right, consistent with our model) or opposed cerebellar splicing patterns (center, inconsistent with our model). Asterisks indicate a significant two-tailed binomial p-value when compared to the fraction of non-changing cerebellar exons that were QKI regulated (cerebellum non-changing versus consistent cerebellum inclusion $-\log_{10}(p) = 10.3$ or versus consistent cerebellum exclusion $-\log_{10}(p) = 10.2$). Non-significant enrichment is indicated by n.s. (versus inconsistent cerebellum inclusion $-\log_{10}(p) = 0.3$ or versus inconsistent cerebellum exclusion $-\log_{10}(p) = 0.3$).

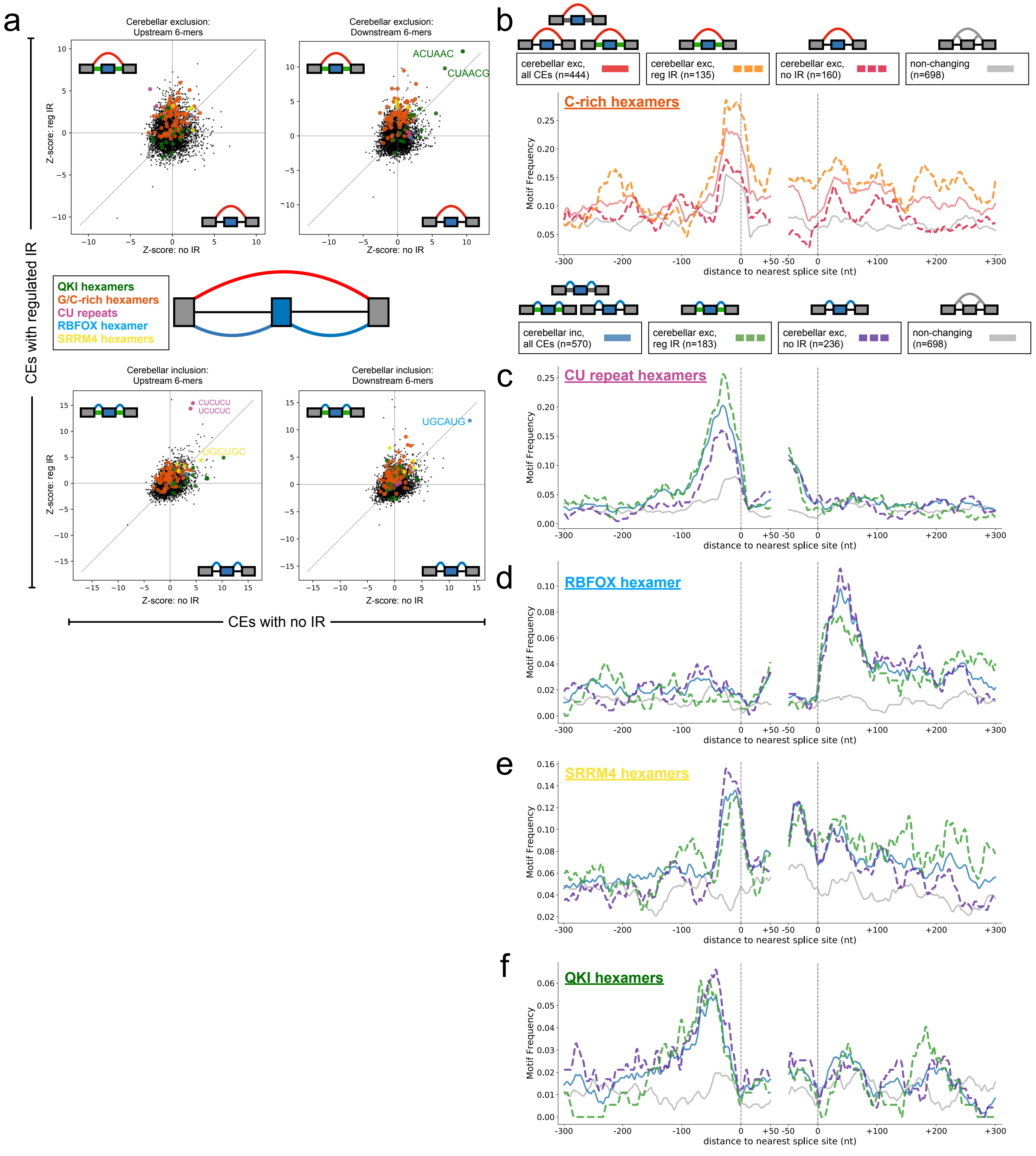
a
Cerebellar exclusion: Upstream 6-mers
Cerebellar exclusion: Downstream 6-mers
ACUAAC
CUAACG
Z-score: reg IR
Z-score: no IR
CEs with regulated IR
QKI hexamers
G/C-rich hexamers
CU repeats
RBFOX hexamer
SRRM4 hexamers
Cerebellar inclusion: Upstream 6-mers
Cerebellar inclusion: Downstream 6-mers
CUCUCU
UCUCUC
UGCUGC
UGCAUG
CEs with no IR
b
cerebellar exc, all CEs (n=444)
cerebellar exc, reg IR (n=135)
cerebellar exc, no IR (n=160)
non-changing (n=698)
C-rich hexamers
Motif Frequency
distance to nearest splice site (nt)
cerebellar inc, all CEs (n=570)
cerebellar exc, reg IR (n=183)
cerebellar exc, no IR (n=236)
non-changing (n=698)
c
CU repeat hexamers
d
RBFOX hexamer
e
SRRM4 hexamers
f
QKI hexamers

Supplementary Fig. 12: Cerebellar cassette exons with and without intron retention. **(a)** Scatter plots showing hexamer Z-score correspondence between non-overlapping sets of cerebellar cassette exon (CE) sets. Each y-axis shows Z-scores from CE events which came from AS modules containing changing intron retention (IR) event(s) versus non-changing. Each x-axis shows Z-scores from CE events coming from AS modules without IR event(s) detected. Motifs of interest are highlighted according to colors in the inset. Top plots show enrichment around cerebellar exclusion event sets while bottom plots show enrichment around cerebellar inclusion event sets. Left plots show Z-scores derived from intronic regions 300 nucleotides upstream of the 3'ss while right plots show Z-scores derived from intronic regions 300 nucleotides downstream of the 5'ss of the cassette exon. All hexamer Z-scores for various CE sets are listed in Table S1. **(b)** RNAmaps for C-rich hexamer motif for given sets of cerebellar exclusion cassette exon event sets. Lines indicate CE set according to the legend: red, all cerebellar exclusion CEs; orange dashed, subset of exclusion CEs which also contained a changing IR event; fuchsia dashed, subset of exclusion CEs with no IR event with the AS module; gray, all CEs which were not changing between comparisons. Frequency of C-rich hexamers (five of six positions are C and contain CCCC) was determined by searching for motif occurrences over sliding windows of 20 nucleotides with smoothing using a running mean of 5 nucleotides. **(c)** RNAmaps for CU-repeat hexamer (CUCUCU,UCUCU) motifs for given sets of cerebellar inclusion cassette exon event sets (plotted as in (b)). Lines indicate CE set according to the legend: blue, all cerebellar inclusion CEs; green dashed, subset of inclusion CEs which also contained a changing IR event; purple dashed, subset of inclusion CEs with no IR event with the AS module; gray, all CEs which were not changing between comparisons. **(d)** Same as in (c), but shown for RBFOX hexamer (UGCAUG). **(e)** Same as in (c), but shown for SRRM4/nSR100 iCLIP hexamers (UGCUGC, CUGCUG, GCUGCC, GCUGCU (*4*)). **(f)** Same as in (c), but shown for QKI hexamers (ACUAAY).

Supplementary References

1. Vaquero-Garcia, J. *et al.* A new view of transcriptome complexity and regulation through the lens of local splicing variations. *elife* **5**, e11752 (2016).

2. Zhang, R., Lahens, N. F., Ballance, H. I., Hughes, M. E. & Hogenesch, J. B. A circadian gene expression atlas in mammals: implications for biology and medicine. *Proceedings of the National Academy of Sciences* **111**, 16219–16224 (2014).

3. Consortium, G. *et al.* The gtex consortium atlas of genetic regulatory effects across human tissues. *Science* **369**, 1318–1330 (2020).

4. Raj, B. *et al.* A global regulatory mechanism for activating an exon network required for neurogenesis. *Molecular cell* **56**, 90–103 (2014).

5. Gonatopoulos-Pournatzis, T. *et al.* Genome-wide crispr-cas9 interrogation of splicing networks reveals a mechanism for recognition of autism-misregulated neuronal microexons. *Molecular cell* **72**, 510–524 (2018).

6. Oberstrass, F. C. *et al.* Structure of ptb bound to rna: specific binding and implications for splicing regulation. *Science* **309**, 2054–2057 (2005).

7. Van Nostrand, E. L. *et al.* A large-scale binding and functional map of human rna-binding proteins. *Nature* **583**, 711–719 (2020).

8. Maticzka, D., Ilik, I. A., Aktas, T., Backofen, R. & Akhtar, A. uvclap is a fast and non-radioactive method to identify in vivo targets of rna-binding proteins. *Nature communications* **9**, 1–13 (2018).
