# Supplementary material for: RNA splicing analysis using heterogeneous and large RNA-seq datasets
Source: Nat Commun. 2023 Mar 3;14:1230. doi: 10.1038/s41467-023-36585-y (PMC9984406; doi:10.1038/s41467-023-36585-y)
Supplement: Supplementary file 3 — Description of Additional Supplementary Files [file 41467_2023_36585_MOESM3_ESM.pdf]

Description of Additional Supplementary Files

File Name: Supplementary Data 1
Description: Hexamer Z scores for cerebellar cassette exon (CE) sets. Z-scores calculated for hexamer occurrence within 300 nucleotides upstream or downstream of cerebellar CE sets versus a set of stringent non-changing cassette exons.

File Name: Supplementary Data 2
Description: Selected samples for performance comparisons on real GTEx data. Selected samples for the different performance comparisons done with real data from GTEx. Each row corresponds to an experiment belonging to a specific comparison between two groups. Column "comparison" indicates which comparison, "group" indicates which of the two groups the experiment belongs to, and "experiment" is the GTEx sample identifier.

File Name: Supplementary Data 3
Description: Selected samples for performance comparisons on simulated GTEx data. Selected samples for the different performance comparisons done with simulated data from GTEx. Each row corresponds to an experiment belonging to a specific comparison between two groups. Column "comparison" indicates which comparison, "group" indicates which of the two groups the experiment belongs to, and "experiment" is the GTEx sample identifier from whose publicly available gene expression quantifications were used to simulate ground-truth RNA-seq reads using BEERS.

File Name: Supplementary Data 4
Description: Tool parameters for performance evaluations. The tools, versions, and additional parameters used for the performance evaluations.

File Name: Supplementary Data 5
Description: Performance evaluation on synthetic data for dPSI 20% at the gene level. All tools evaluated using the simulated GTEx data on reporting events with dPSI of at least 20%, using sample group sizes between 3 and 50. Events are summarized at the level of genes.

File Name: Supplementary Data 6
Description: Performance evaluation on synthetic data for dPSI 10% at the gene level. All tools evaluated using the simulated GTEx data on reporting events with dPSI of at least 10%, using sample group sizes between 3 and 50. Events are summarized at the level of genes.

File Name: Supplementary Data 7
Description: Performance evaluation on synthetic data for dPSI 20% at the event level. All tools evaluated using the simulated GTEx data on reporting events with dPSI of at least 20%, using sample group sizes between 3 and 50.

File Name: Supplementary Data 8
Description: Performance evaluation on synthetic data for dPSI 10% at the event level. All tools evaluated using the simulated GTEx data on reporting events with dPSI of at least 10%, using sample group sizes between 3 and 50.
