# Supplementary material for: RNA splicing analysis using heterogeneous and large RNA-seq datasets
Source: Nat Commun. 2023 Mar 3;14:1230. doi: 10.1038/s41467-023-36585-y (PMC9984406; doi:10.1038/s41467-023-36585-y)
Supplement: Supplementary file 12 — Reporting Summary [file 41467_2023_36585_MOESM12_ESM.pdf]

nature portfolio

Corresponding author(s): Yoseph Barash

Last updated by author(s): Jan 4, 2022

Reporting Summary

Nature Portfolio wishes to improve the reproducibility of the work that we publish. This form provides structure for consistency and transparency in reporting. For further information on Nature Portfolio policies, see our Editorial Policies and the Editorial Policy Checklist.

Statistics

For all statistical analyses, confirm that the following items are present in the figure legend, table legend, main text, or Methods section.

n/a	Confirmed	
☐	☒	The exact sample size (*n*) for each experimental group/condition, given as a discrete number and unit of measurement
☐	☒	A statement on whether measurements were taken from distinct samples or whether the same sample was measured repeatedly
☐	☒	The statistical test(s) used AND whether they are one- or two-sided<br>*Only common tests should be described solely by name; describe more complex techniques in the Methods section.*
☐	☒	A description of all covariates tested
☐	☒	A description of any assumptions or corrections, such as tests of normality and adjustment for multiple comparisons
☐	☒	A full description of the statistical parameters including central tendency (e.g. means) or other basic estimates (e.g. regression coefficient) AND variation (e.g. standard deviation) or associated estimates of uncertainty (e.g. confidence intervals)
☐	☒	For null hypothesis testing, the test statistic (e.g. *F*, *t*, *r*) with confidence intervals, effect sizes, degrees of freedom and *P* value noted<br>*Give P values as exact values whenever suitable.*
☒	☐	For Bayesian analysis, information on the choice of priors and Markov chain Monte Carlo settings
☒	☐	For hierarchical and complex designs, identification of the appropriate level for tests and full reporting of outcomes
☐	☒	Estimates of effect sizes (e.g. Cohen's *d*, Pearson's *r*), indicating how they were calculated

Our web collection on statistics for biologists contains articles on many of the points above.

Software and code

Policy information about availability of computer code

Data collection	SRATools (v2.9.6) for downloading dbGaP data from NCBI SRA, gsutil (v4.46) for downloading GTEx data from Google Cloud hosting when applicable, BEERS algorithm (http://cbil.upenn.edu/BEERS/) to generate simulated RNA-Seq data.
Data analysis	SRATools (v2.9.6)<br>Samtools (v1.9)<br>BEERS (http://cbil.upenn.edu/BEERS/)<br>TrimGalore (v0.4.5)<br>STAR (v2.5.3a)<br>Salmon (v0.14.0)<br>MAJIQ (v2.3)<br>rMATS-turbo (v4.1.0)<br>LeafCutter (git hash 2440827)<br>SUPPA2 (v2.3)<br>Whippet (v1.0)

For manuscripts utilizing custom algorithms or software that are central to the research but not yet described in published literature, software must be made available to editors and reviewers. We strongly encourage code deposition in a community repository (e.g. GitHub). See the Nature Portfolio guidelines for submitting code & software for further information.

March 2021

Data

Policy information about availability of data

All manuscripts must include a data availability statement. This statement should provide the following information, where applicable:

- Accession codes, unique identifiers, or web links for publicly available datasets
- A description of any restrictions on data availability
- For clinical datasets or third party data, please ensure that the statement adheres to our policy

The raw simulated RNA-seq data for cerebellum and skeletal muscle generated in this study have been deposited in the GEO database under accession code GSE222044 at https://www.ncbi.nlm.nih.gov/geo/query/acc.cgi?acc=GSE222044. Raw GTEx data used for the analyses in this manuscript are available in dbGaP under accession code phs000424.v8.p2 at https://www.ncbi.nlm.nih.gov/projects/gap/cgi-bin/study.cgi?study_id=phs000424.v8.p2. ENCODE raw RNA-seq for QKI knockdown and eCLIP peaks were downloaded from https://www.encodeproject.org/ under accession codes ENCSR366YOG, ENCSR570WLM, and ENCSR330YOU. Processed data and code to reproduce figures have been deposited in a Zenodo repository (DOI 10.5281/zenodo.7508313) available at https://doi.org/10.5281/zenodo.7508313.

Field-specific reporting

Please select the one below that is the best fit for your research. If you are not sure, read the appropriate sections before making your selection.

☒ Life sciences ☐ Behavioural & social sciences ☐ Ecological, evolutionary & environmental sciences

For a reference copy of the document with all sections, see nature.com/documents/nr-reporting-summary-flat.pdf

Life sciences study design

All studies must disclose on these points even when the disclosure is negative.

Sample size	We selected 150 samples per tissue for the comparisons between cerebellum and muscle (both real and simulated data cases) in order to show a range of sample size cases. For the brain subregions analysis, we selected all brain tissue samples that satisfied our requirements on RIN.
Data exclusions	We required all samples to have a RIN score of greater than 6 to avoid technical artifacts from analyzing degraded samples.
Replication	Data and associated scripts to reproduce the results are with the source code for MAJIQ v2.
Randomization	Selection of samples for analysis per tissue was done randomly from available data when taking subsets.
Blinding	Blinding is not applicable because we used previously generated data from other studies or simulated the data based on existing samples from previous studies

Reporting for specific materials, systems and methods

We require information from authors about some types of materials, experimental systems and methods used in many studies. Here, indicate whether each material, system or method listed is relevant to your study. If you are not sure if a list item applies to your research, read the appropriate section before selecting a response.

Materials & experimental systems

n/a	Involved in the study
☒	☐ Antibodies
☒	☐ Eukaryotic cell lines
☒	☐ Palaeontology and archaeology
☒	☐ Animals and other organisms
☒	☐ Human research participants
☒	☐ Clinical data
☒	☐ Dual use research of concern

Methods

n/a	Involved in the study
☒	☐ ChIP-seq
☒	☐ Flow cytometry
☒	☐ MRI-based neuroimaging
